# Dietary modifications affect renal recovery during the healing phase following ischemic acute ischemic kidney injury

**DOI:** 10.3389/fcell.2025.1494660

**Published:** 2025-05-22

**Authors:** Junseok Jeon, Kyungho Lee, Hojin Jeon, Kyeong Eun Yang, Cheol-Jung Lee, Jung Eun Lee, Ghee Young Kwon, Wooseong Huh, Hye Ryoun Jang

**Affiliations:** ^1^ Division of Nephrology, Department of Medicine, Samsung Medical Center, Cell and Gene Therapy Institute, Sungkyunkwan University School of Medicine, Seoul, Republic of Korea; ^2^ Division of Scientific Instrumentation & Management, Korea Basic Science Institute, Daejeon, Republic of Korea; ^3^ Department of Pathology, Samsung Medical Center, Sungkyunkwan University School of Medicine, Seoul, Republic of Korea

**Keywords:** acute kidney injury, healing, diet, immunologic micromilieu, ischemia-reperfusion injury

## Abstract

**Introduction:**

The effects of dietary modifications, such as varying amounts of salt, fat, and protein intake on the healing phase of acute kidney injury (AKI) remain to be elucidated. We investigated the effects of low- or high-salt/fat/protein diets on the intrarenal immunologic micromilieu and healing after renal ischemia-reperfusion injury (IRI) using murine ischemic AKI and human kidney-2 (HK-2) cell hypoxia models.

**Methods:**

Three cohorts of male C57BL/6 mice (9-weeks old) were fed the designated diet from the third day following renal IRI until sacrifice (6 or 12 weeks after bilateral or unilateral IRI, respectively) in groups as follows: cohort 1, control, high- and low-salt/fat/protein; cohort 2, control, high- and low-salt; cohort 3, control, high- and low-fat/protein. Hypoxic HK-2 cells were treated with sodium chloride, amino acids, or fatty acids.

**Results:**

Low-salt/fat/protein diet aggravated interstitial fibrosis, enhanced TGF-β expression, and induced more proinflammatory changes after bilateral IRI. High-salt diet aggravated renal tubular damage and enhanced the expression of intrarenal TGF-β after bilateral IRI, whereas low-salt diet enhanced the expression of intrarenal TGF-β after unilateral IRI. Low-salt diet induced more proinflammatory changes after bilateral IRI. Blood urea nitrogen levels were lower in the low fat/protein group than that in the control group following IRI. However, low-fat/protein diet aggravated interstitial fibrosis and enhanced intrarenal TGF-β expression after unilateral IRI. High sodium- or protein-containing media suppressed the proliferation of hypoxic HK-2 cells, whereas high lipid-containing media enhanced the proliferation of hypoxic HK-2 cells.

**Conclusion:**

Excessive low or high salt, low fat, and low protein diet may adversely affect the healing process following renal IRI, supporting the importance of adequate and balanced nutrition during the recovery phase of ischemic AKI.

## 1 Introduction

Dietary composition affects the immune system (Christ et al., 2019). Dietary modification alters disease severity in salt-sensitive hypertension or high fat-induced obesity models, partly because of its effects on immune mechanisms (Laurentius et al., 2019; Mattson, 2019). In a previous study, we demonstrated that high-fat or high-salt diet increased intrarenal CD8^+^ T and plasma cells, and upregulated intrarenal proinflammatory cytokines, such as TNF-α and MCP-1 in normal mouse kidneys. These immunological changes increased the susceptibility of the mice to early renal injury following bilateral ischemia-reperfusion injury (IRI) (Jeon et al., 2021).

Ischemic acute kidney injury (AKI) is the most common cause of AKI, and often contributes to the development and progression of chronic kidney disease (CKD) (Chawla et al., 2014; Nash et al., 2002). IRI leads to the paradoxical worsening of cellular dysfunction and tissue damage after the restoration of blood flow into the ischemic tissue (Chawla et al., 2014). The process of renal injury in ischemic AKI is more complex than that in hypoxic injury, and is mediated by immunological mechanisms involving both the innate and adaptive immune systems (Donnahoo et al., 1999; Furuichi et al., 2003; Jang et al., 2009). Individual variations in the length of the healing phase and the degree of recovery remain serious challenges in patients with ischemic AKI as no specific treatment option is currently available.

Our previous study focused on the effects of dietary modifications on normal mouse kidneys and the early injury phase of ischemic AKI (Jeon et al., 2021). However, the effects of dietary modifications on the healing phase following ischemic AKI remains unclear. Dietary modification represents an attractive therapeutic strategy that can be easily incorporated into clinical practice. Therefore, in the present study we sought to investigate the effects of dietary modifications during the healing phase following ischemic AKI on post-ischemic intrarenal immunologic micromilieu and renal recovery.

## 2 Materials and methods

### 2.1 Dietary modifications and the renal IRI model

Male C57BL/6 mice (9-weeks old) were purchased from Orient Bio Inc. (Seongnam, Kyoungki-do, Korea) and housed in a pathogen-free facility. A previously established murine IRI model using a laparotomy approach was used as the ischemic AKI model (Jang et al., 2009; Jang et al., 2014). Briefly, the mice were anesthetized with intraperitoneal injections of ketamine (100 mg/kg; Yuhan, Seoul, Korea) and xylazine (10 mg/kg; Bayer, Leverkusen, Germany). The anesthetized mice were placed on a thermostatically controlled heating table and maintained hydrated with warm sterile saline during the procedure. Following an abdominal midline incision, both (bilateral model) or left (unilateral) renal pedicles were isolated and clamped using a microvascular clamp (Roboz Surgical Instruments, Gaithersburg, MD, United States). The clamps were removed from the renal pedicles for reperfusion after 27 min for the bilateral model and 45 min for the unilateral model. Following suturing, the mice were provided unrestricted access to their allocated food and water during the healing period. Three days after IRI, the mice were fed the designated diet in three cohorts as follows until sacrifice: cohort 1 – control, high salt/fat/protein, and low salt/fat/protein; cohort 2 – control, high salt, and low salt; cohort 3 – control, high fat/protein, and low fat/protein. The detailed dietary compositions are provided in Table 1. The mice were sacrificed 2, 4, or 6 weeks after bilateral IRI and 6 or 12 weeks after unilateral IRI, and the kidneys were harvested after exsanguination.

**TABLE 1:** Dietary composition of the studied diet.

Diet group	Control	High salt/fat/protein	Low salt/fat/protein	High salt	Low salt	High fat/protein	Low fat/protein
Protein, kcal%	25	28	3	21	21	28	3
Fat, kcal%	13	42	7	12	12	42	7
Carbohydrate, kcal%	62	30	90	68	68	30	90
NaCl, weight%	0.8	8.2	0.3	7.6	0.3	0.8	0.8

### 2.2 Assessment of renal function and blood pressure

Blood urea nitrogen (BUN; Fujifilm, Bedford, United Kingdom) and creatinine (Arbor Assays, Ann Arbor, MI) concentrations were measured in plasma samples from mice using colorimetric kits according to the manufacturer’s instructions. Blood pressure was measured in conscious mice by tail-cuff method using an automated cuff inflator-pulse detection system (Kent Scientific Corporation, CT, United States).

### 2.3 Histological analysis of tissues

Post-ischemic kidney tissue sections were fixed in 10% buffered-formalin and stained with hematoxylin and eosin (H&E). The percentage of renal tubular necrosis, damage, or atrophy of the entire renal tubule was assessed by a renal pathologist blinded to the diet assignment.

### 2.4 CD45 immunohistochemistry and tissue FAXS analysis

Formalin-fixed sections of renal tissues were immunostained for CD45 as previously described (Jang et al., 2014). The 4-µm-thick sections were deparaffinized with xylene, rehydrated using a succession of graded alcohol, and then treated with citrate buffer (pH 6.0). To enhance antigen retrieval, the slides were heated in a pressure cooker for 10 min using microwaves. The sections were allowed to cool, incubated with Serum-Free Protein Block (Dako, Carpinteria, CA, United States) overnight at 4°C, and then immersed in hydrogen peroxide solution (Dako) for 30 min to inhibit endogenous peroxidase activity. The following day, the slides were treated with anti-mouse CD45 monoclonal antibody (1:100; BD Biosciences, San Jose, CA, United States) for 1 h at room temperature. The CD45-stained sections were rinsed, and then incubated with the secondary antibody using a Dako REAL EnVision Kit (Dako) for 30 min at room temperature. The slides were counterstained with Mayer’s hematoxylin solution (Dako) after treatment with 3,3′-diaminobenzidine tetrahydrochloride (Dako). A TissueFAXS workstation (TissueGnostics, Vienna, Austria) was used to examine the sections and determine the proportion of CD45-positive cells in the kidney samples, as previously described (Jang et al., 2014).

### 2.5 Flow cytometric analysis of kidney-infiltrating mononuclear cells (KMNCs)

KMNCs were isolated using a previously established protocol (Ascon et al., 2006). Briefly, decapsulated kidneys were mechanically disrupted in RPMI buffer (Mediatech, Manassas, VA, United States) containing 5% fetal bovine serum using a Stomacher® 80 Biomaster (Seward, Worthing, United Kingdom). Samples were strained, washed, and resuspended in 36% Percoll (Amersham Pharmacia Biotech, Piscataway, NJ, United States), followed by gentle overlaying on 72% Percoll. Subsequently, the samples were centrifuged at 1,000 × g for 30 min at room temperature. KMNCs were collected from the interface of the 36% and 72% Percoll.

The isolated KMNCs were resuspended in fluorescence-activated cell sorting (FACS) buffer and pre-incubated with anti-CD16/CD32 antibodies for 10 min to minimize nonspecific binding through Fc receptors. The cells were then incubated with anti-mouse CD3, CD4, CD8, CD19, CD21, CD25, CD44, CD45, CD62L, CD69, CD126, CD138, Gr-1, F4/80, FoxP3, and NK1.1 antibodies (all from BD Biosciences) for 25 min at 4°C. The cells were washed with FACS buffer and fixed with 1% paraformaldehyde solution. The samples were analyzed using a BD FACSVerse™ flow cytometer (BD Biosciences). Data were analyzed using the FlowJo software (version 10.10.0, BD Biosciences).

### 2.6 Multiplex cytokine/chemokine assay

Multiplex cytokine and chemokine analyses were performed on whole kidney protein extracts using a Milliplex® MAP Mouse Cytokine/Chemokine Kit (Luminex, Austin, TX, United States) according to the manufacturer’s instructions. This multiplexed particle-based flow cytometric technique employs anti-cytokine monoclonal antibodies linked to microspheres integrating the distinct properties of the two fluorescent dyes. The assay was used to measure the following cytokines/chemokines: interleukin (IL)-2, IL-4, IL-6, IL-10, interferon (IFN)-γ, monocyte chemoattractant protein (MCP)-1, regulated on activation, normal T cell expressed and secreted (RANTES/CCL5), tumor necrosis factor (TNF)-α, and vascular endothelial growth factor (VEGF). The value for each cytokine or chemokine was normalized by dividing the raw concentration (pg/mL) by the kidney protein concentration (mg/mL) measured using the Pierce® BCA Protein Assay Kit (Thermo Fisher Scientific, Waltham, MA, United States).

### 2.7 HK-2 cell hypoxia model and proliferation assay

An HK-2 cell (an immortalized proximal tubule epithelial cell line from a normal adult human kidney) hypoxia model was used as an *in vitro* model to study the effects of dietary modifications on healing at the cellular level post-hypoxia. HK-2 cells were purchased from American Type Culture Collection (CRL-2190, Manassas, VA, United States) and cultured in keratinocyte serum-free medium (Thermo Fisher Scientific) supplemented with bovine pituitary extract and human recombinant epidermal growth factor. Cells were incubated at 37°C in a humidified atmosphere with 5% CO_2_ and the medium was changed every 2–3 days. Hypoxia was induced by exposing the cells to 1% O_2_ and 5% CO_2_ (balanced with nitrogen) in a multi-gas incubator (APM-30D; Astec, Fukuoka, Japan) for 48 h. Following hypoxia treatment, the cells were exposed to different concentrations of sodium, fatty acids, and amino acids. HK-2 cell proliferation after hypoxia was assessed using a CellTiter 96® AQueous One Solution Cell Proliferation Assay Kit (Promega, Madison, WI, United States) according to the manufacturer’s instructions.

### 2.8 Statistical analyses

All data are expressed as the mean ± standard error of the mean. Differences between groups were analyzed using one-way analysis of variance followed by Tukey’s *post hoc* analysis. All statistical analyses were performed using the GraphPad Prism software (version 10, GraphPad Software Inc., Boston, MA, United States). Statistical significance was set at *P* < 0.05.

## 3 Results

### 3.1 Effects of high- or low-salt/fat/protein diets on physiological changes, tissue damage, and fibrosis following renal IRI

**FIGURE 1:**
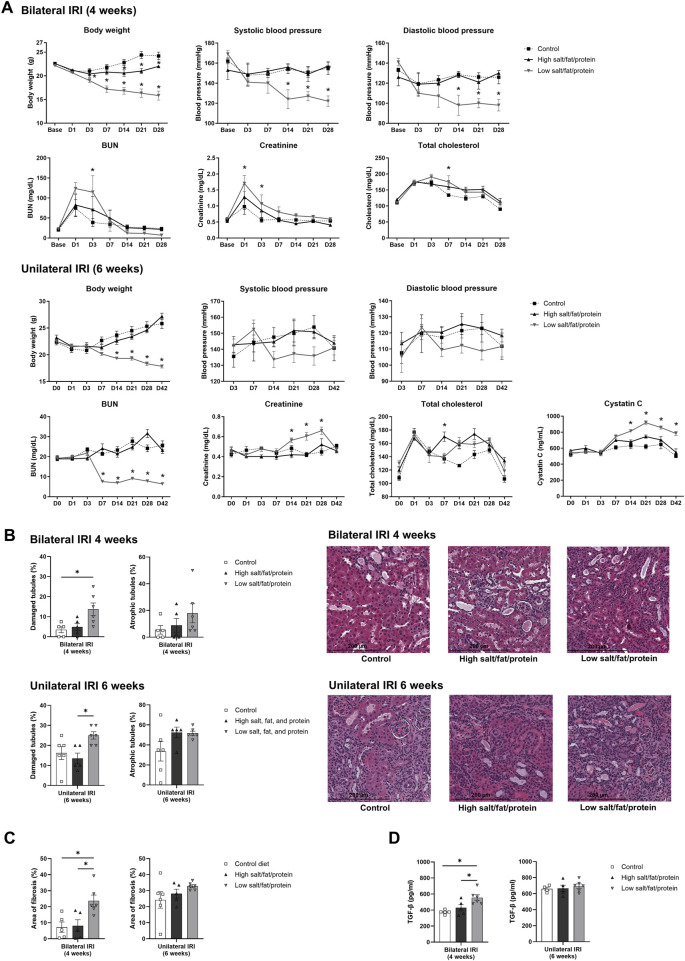
Changes in renal function, tubular injury, and fibrosis in mice fed high- or low-salt/fat/protein diet after renal ischemia-reperfusion injury. **(A)** Changes in body weight, blood pressure, renal function, and total cholesterol. **(B)** Tubular damage or atrophy. **(C)** Degree of interstitial fibrosis. **(D)** Intrarenal expression of TGF-β. *P < 0.05. Statistical analyses were performed using analysis of variance test followed by Tukey’s test.

Changes in body weight, blood pressure, and renal function following renal IRI in mice fed high- or low-salt/fat/protein diets are shown in Figure 1A. The high-salt/fat/protein diet group tended to have a lower body weight than the control group, although the blood pressure of the high-salt/fat/protein diet group was not different from that of the control group. The low-salt/fat/protein diet group exhibited a significantly lower body weight and blood pressure than the control group. Following bilateral IRI, no differences were noted in plasma creatinine or BUN levels between the control and high-salt/fat/protein diet groups. The low-salt/fat/protein diet group showed higher plasma creatinine and BUN levels shortly after bilateral IRI, but no difference was observed after the seventh day. After unilateral IRI, the low-salt/fat/protein diet group showed lower BUN, but higher cystatin C and creatinine levels than the control and high-salt/fat/protein diet groups.

The extent of renal tubular damage and atrophy, tissue fibrosis, and intrarenal TGF-β expression following renal IRI in mice fed various diets is shown in Figures 1B–D. Following bilateral IRI, renal tubular damage was aggravated with enhanced fibrosis and increased TGF-β expression in the low-salt/fat/protein diet group than that in the control and high-salt/fat/protein groups. Following unilateral IRI, the extent of renal tubular damage and atrophy, tissue fibrosis, and TGF-β expression was comparable among the groups.

### 3.2 Effects of high- or low-salt/fat/protein diets on the intrarenal immunologic micromilieu following renal IRI

**FIGURE 2:**
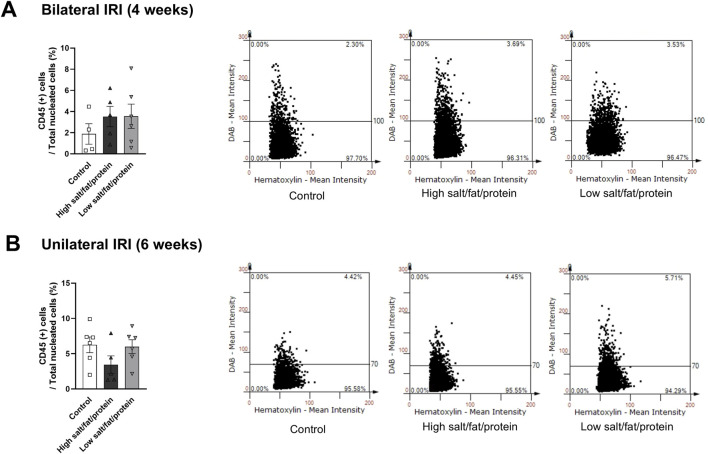
Leukocyte trafficking into the post-ischemic kidneys in mice fed low- or high-salt/protein/fat diet. Proportion of intrarenal total leukocytes expressing CD45 among total nuclei in the whole field after **(A)** bilateral and **(B)** unilateral ischemia-reperfusion injury. *P < 0.05. Statistical analyses were performed using analysis of variance test followed by Tukey’s test. Activated T cell, CD69^+^ subset; effector memory T cells, CD44^+^ CD62L-subset.

**FIGURE 3:**
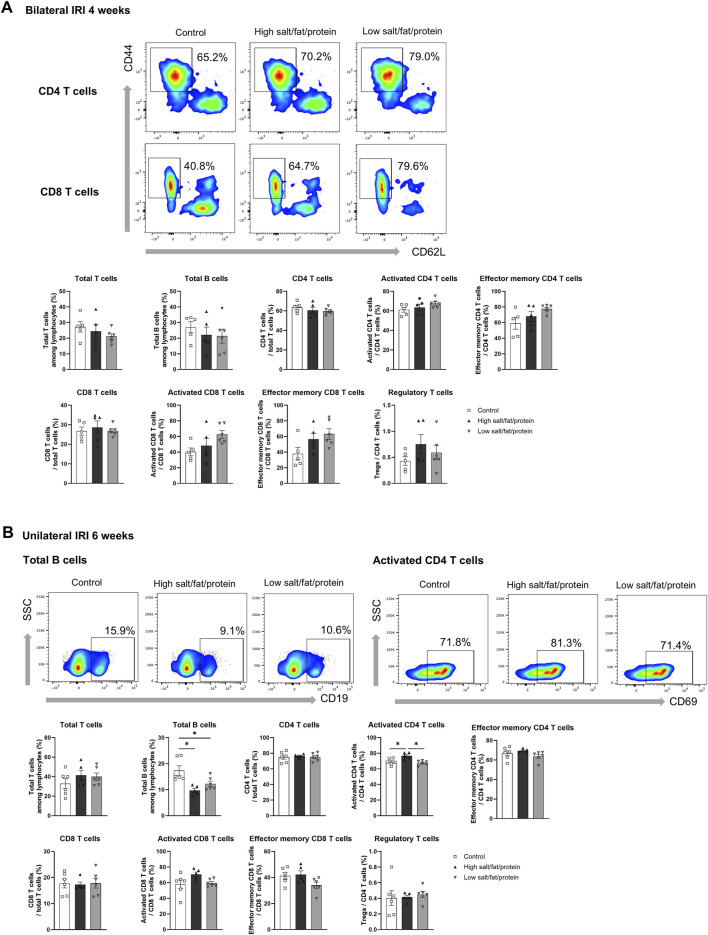
Intrarenal leukocyte subtypes in the post-ischemic kidneys in mice fed low- or high-salt/protein/fat diet. Intrarenal leukocyte subtypes after **(A)** bilateral and **(B)** unilateral ischemia-reperfusion injury. *P < 0.05. Statistical analyses were performed using analysis of variance test followed by Tukey’s test. Activated T cells, CD69^+^ subset; Effector memory T cells, CD44^+^ CD62L-subset; Regulatory CD4 T cells, FoxP3+ CD25^+^ CD4 T cell subset.

The proportion of total intrarenal leukocytes expressing CD45 among all the cells in the whole field in each slide was semi-quantitatively calculated using the TissueFAXS system (Figure 2). Intrarenal total leukocytes expressing CD45 did not differ between the high- or low-salt/fat/protein diet groups following bilateral or unilateral IRI. The intrarenal leukocyte subtypes after renal IRI in mice fed high- or low-salt/fat/protein diet are shown in Figures 3A,B. Following bilateral IRI, the proportion of effector memory CD4^+^ T (P = 0.075), activated CD8^+^ T (P = 0.071), and effector memory CD8^+^ T (P = 0.067) cells was tended to be higher in the low-salt group than that in the control group. Following unilateral IRI, the proportion of total B cells was lower in the high- and low-salt/fat/protein groups compared with that in the control group, and the proportion of activated CD4^+^ T cells was higher in the high-salt/fat/protein diet group compared with that in the control and low-salt/fat/protein diet groups. The proportion of B cell subtypes, neutrophils, macrophages, and NK cells following IRI was comparable between the groups.

**FIGURE 4:**
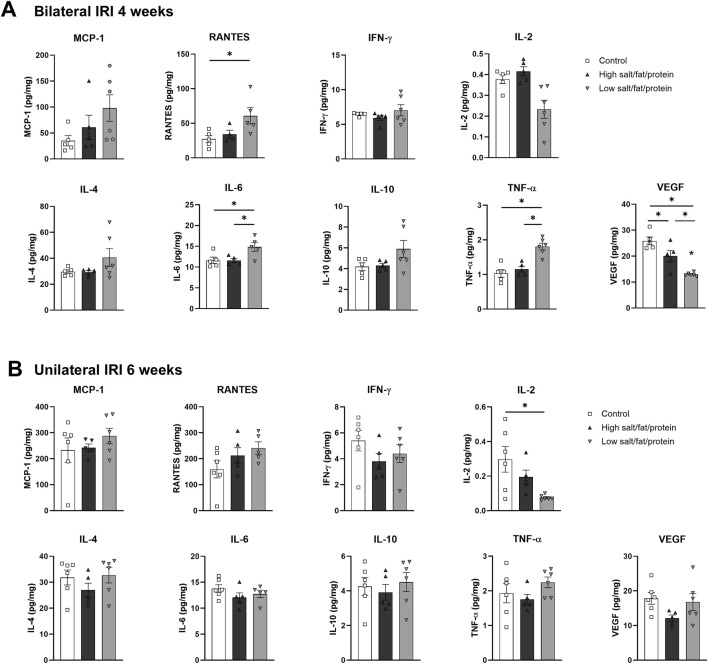
Expression of intrarenal cytokines and chemokines in the post-ischemic kidneys in mice fed low- or high-salt/protein/fat diet. Expression of intrarenal cytokines and chemokines after **(A)** bilateral and **(B)** unilateral ischemia-reperfusion injury. *P < 0.05. Statistical analyses were performed using analysis of variance test followed by Tukey’s test.

Intrarenal cytokine/chemokine levels in mice following renal IRI according to the diet type are shown in Figure 4. Following bilateral IRI, the concentrations of RANTES, IL-6, and TNF-α were higher, whereas that of VEGF was lower at 4 weeks in the low-salt/fat/protein group compared with that in the control and high-salt/fat/protein groups (Figure 4A). Following unilateral IRI, IL-2 levels were lower in the low-salt/fat/protein group compared with that in the control group (Figure 4B).

### 3.3 Effects of high- or low-salt diets on physiological changes, tissue damage, and fibrosis following renal IRI

**FIGURE 5:**
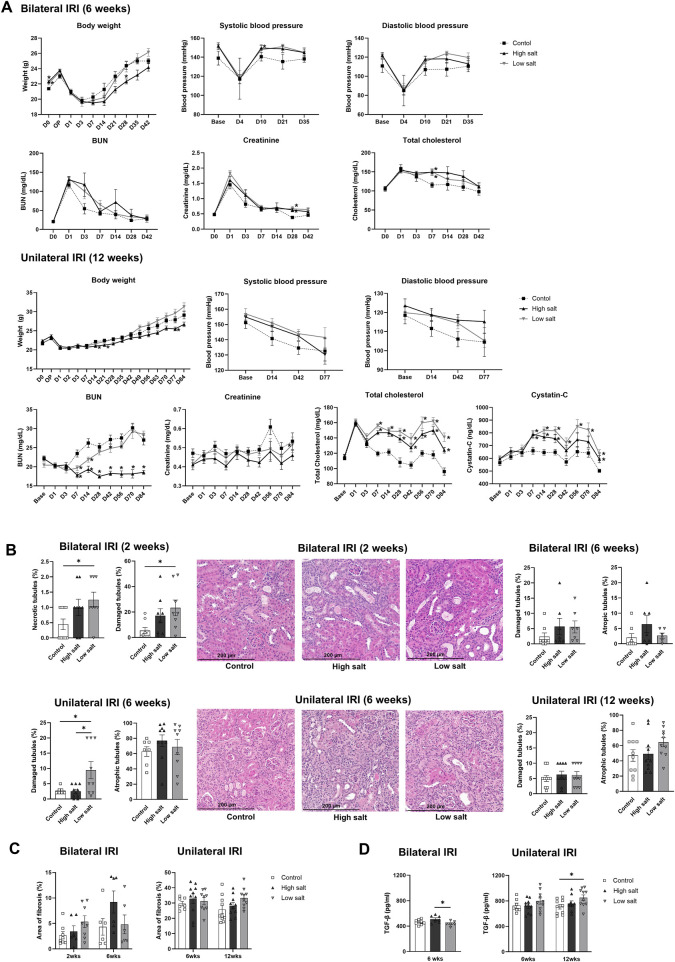
Changes in renal function, tubular injury, and fibrosis in mice fed low or high-salt diet after renal ischemia-reperfusion injury. **(A)** Changes in body weight, blood pressure, renal function, and total cholesterol. **(B)** Tubular necrosis, damage, or atrophy. **(C)** Degree of interstitial fibrosis. **(D)** Intrarenal expression of TGF-β. *P < 0.05. Statistical analyses were performed using analysis of variance test followed by Tukey’s test.

Changes in body weight, blood pressure, and renal function following renal IRI in mice fed high- or low-salt diet are shown in Figure 5A. The high-salt diet group tended to have a lower body weight than the control group after renal IRI. However, no difference in blood pressure was observed between the groups. BUN levels were lower in the high salt group than that in the control group after unilateral IRI. However, plasma creatinine levels did not differ between the groups following bilateral and unilateral IRI. Plasma total cholesterol and cystatin C levels were higher in both high- and low-salt diet groups compared with that in the control group after unilateral IRI.

The extent of renal tubular damage and atrophy, interstitial fibrosis, and intrarenal TGF-β expression following renal IRI in mice fed high- or low-salt diet are shown in Figures 5B–D. The proportion of necrotic and damaged tubules was higher in the low salt group than that in the control group at 2 weeks, and the proportion of damaged tubules was higher in the low salt group than that in the control and high salt groups at 6 weeks following bilateral IRI (Figure 5B). However, no difference in tubular damage or atrophy was noted between the groups at 12 weeks after unilateral IRI. In addition, there was no significant difference in tissue fibrosis between the groups after bilateral and unilateral IRI. Intrarenal TGF-β expression was higher in the high salt group than that in the low salt group at 6 weeks after bilateral IRI. Similarly, TGF-β expression was higher in the low salt group than that in the control group at 12 weeks after unilateral IRI.

### 3.4 Effects of high- or low-salt diet on the intrarenal immunologic micromilieu following renal IRI

**FIGURE 6:**
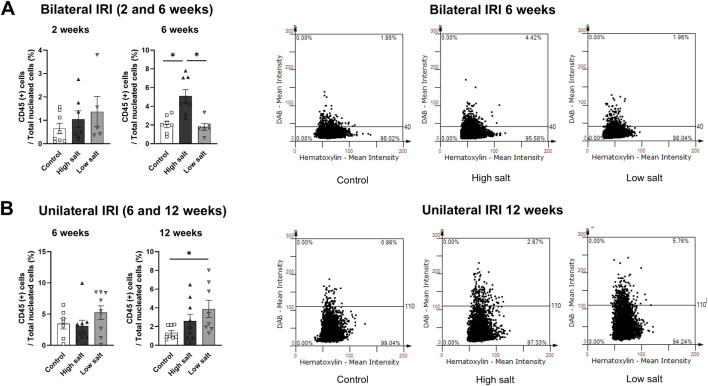
Leukocyte trafficking into the post-ischemic kidneys in mice fed low- or high-salt diet. Proportion of intrarenal total leukocytes expressing CD45 among total nuclei in the whole field after **(A)** bilateral and **(B)** unilateral ischemia-reperfusion injury. *P < 0.05. Statistical analyses were performed using analysis of variance test followed by Tukey’s test.

**FIGURE 7:**
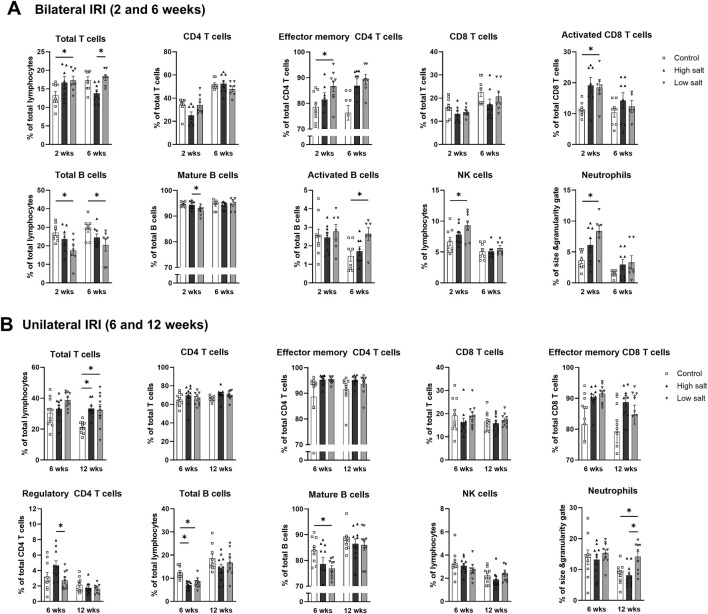
Intrarenal leukocyte subtypes in the post-ischemic kidneys in mice fed low- or high-salt diet. Intrarenal leukocyte subtypes after **(A)** bilateral and **(B)** unilateral ischemia-reperfusion injury. *P < 0.05. Statistical analyses were performed using analysis of variance test followed by Tukey’s test. Activated T cell, CD69^+^ subset; effector memory T cells, CD44^+^ CD62L-subset.

The high salt group exhibited higher proportion of intrarenal total leukocytes expressing CD45 compared with the control and low salt groups 6 weeks after bilateral IRI (Figure 6A). In contrast, the low salt group showed higher proportion of total intrarenal leukocytes expressing CD45 than the control group after unilateral IRI (Figure 6B). Intrarenal leukocyte subtypes following renal IRI in groups fed high- or low-salt diet are shown in Figure 7. Following bilateral IRI, the post-ischemic kidneys of the low salt group contained a higher proportion of total T cells, effector memory CD4 T cells, activated CD8 T cells, activated B cells, NK cells, and neutrophils, and a lower proportion of total B cells than the control group (Figure 7A). After unilateral IRI, the low salt group had a higher proportion of total T cells and neutrophils, and a lower proportion of total and mature B cells than the control group (Figure 7B). The high salt group had a higher proportion of total T cells and a lower proportion of total B cells than the control group. Furthermore, the high salt group had a higher proportion of regulatory T cells and a lower proportion of neutrophils compared with the low salt group.

**FIGURE 8:**
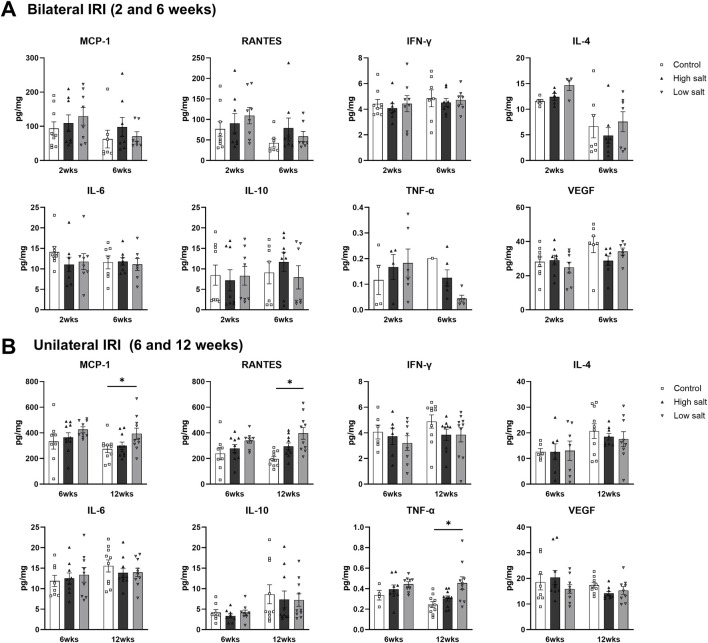
Expression of intrarenal cytokines and chemokines in the post-ischemic kidneys in mice fed low- or high-salt diet. Expression of intrarenal cytokines and chemokines after **(A)** bilateral and **(B)** unilateral ischemia-reperfusion injury. *P < 0.05. Statistical analyses were performed using analysis of variance test followed by Tukey’s test.

The intrarenal cytokine/chemokine levels in mice after renal IRI according to a high- or low-salt diet are shown in Figure 8. The concentration of intrarenal cytokine/chemokine after bilateral IRI was comparable between the groups (Figure 8A). After unilateral IRI, the levels of MCP-1, RANTES, and TNF-α were higher in the low salt group than that in the control group at 12 weeks (Figure 8B).

### 3.5 Effects of high- or low-fat/protein diets on physiological changes, tissue damage, and fibrosis following renal IRI

**FIGURE 9:**
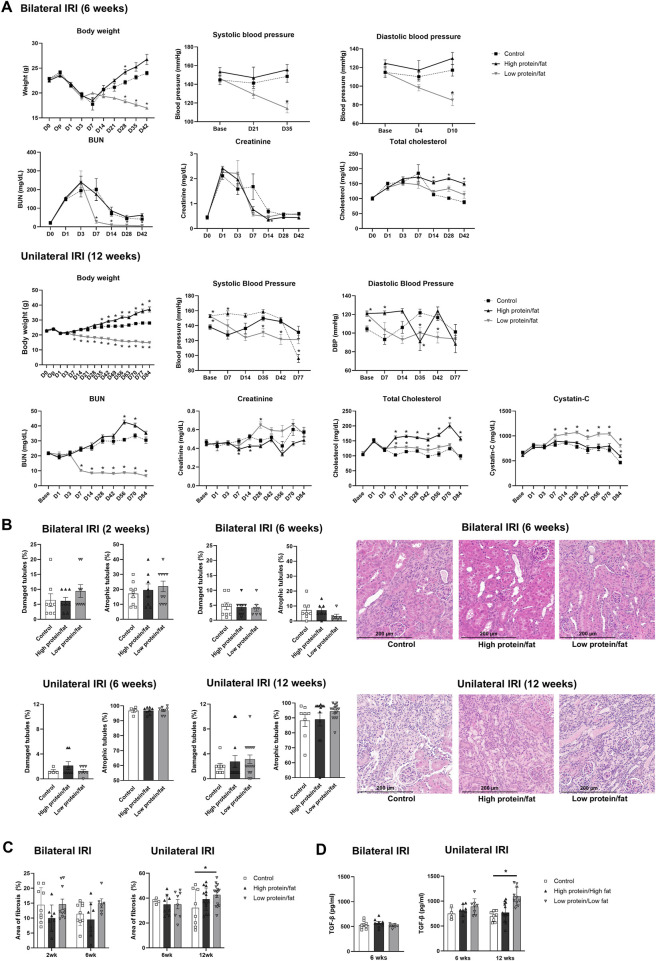
Changes in renal function, tubular injury, and fibrosis in mice fed high- or low-fat/protein diets after renal ischemia-reperfusion injury. **(A)** Changes in body weight, blood pressure, renal function, and total cholesterol. **(B)** Tubular necrosis, damage, or atrophy. **(C)** Degree of interstitial fibrosis. **(D)** Intrarenal expression of TGF-β. *P < 0.05. Statistical analyses were performed using analysis of variance test followed by Tukey’s test.

Changes in body weight, blood pressure, and renal function following renal IRI in groups fed high- or low-fat/protein diets are shown in Figure 9A. The high-fat/protein group had higher body weight, whereas the low-fat/protein group had lower body weight compared with the control group post IRI. In addition, the high-fat/protein group tended to have higher blood pressure and the low-fat/protein group tended to have lower blood pressure compared with the control group. Plasma creatinine levels were comparable between the groups, but BUN levels were lower in the low-fat/protein group than that in the control group after IRI. The high-fat/protein group showed higher total cholesterol levels than the control group after IRI. After unilateral IRI, cystatin C levels were higher in the low-fat/protein group than that in the control group.

The extent of renal tubular damage and atrophy, interstitial fibrosis, and intrarenal TGF-β expression following renal IRI in groups fed high- or low-fat/protein diets are shown in Figures 9B–D. No differences were observed in tubular damage or atrophy between the groups after IRI (Figure 9B). The degree of interstitial fibrosis was significantly more severe in the low-fat/protein group than that in the control group at 12 weeks after unilateral IRI (Figure 9C). However, the degree of interstitial fibrosis was comparable between the high-fat/protein and control groups. Intrarenal TGF-β expression was higher in the low-fat/protein group than that in the control group at 12 weeks after unilateral IRI (Figure 9D).

### 3.6 Effects of high- or low-fat/protein diets on the intrarenal immunologic micromilieu after renal IRI

**FIGURE 10:**
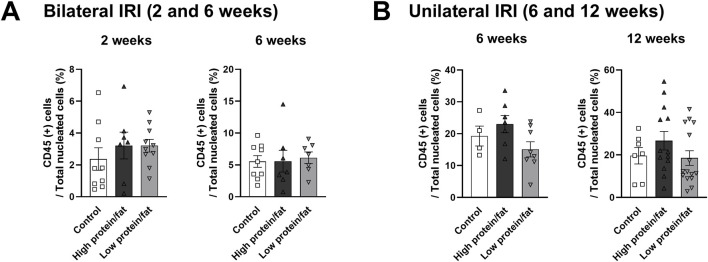
Leukocyte trafficking into the post-ischemic kidneys in mice fed low- or high-fat/protein diets. Proportion of intrarenal total leukocytes expressing CD45 among total nuclei in the whole field after **(A)** bilateral and **(B)** unilateral ischemia-reperfusion injury. *P < 0.05. Statistical analyses were performed using analysis of variance test followed by Tukey’s test.

**FIGURE 11:**
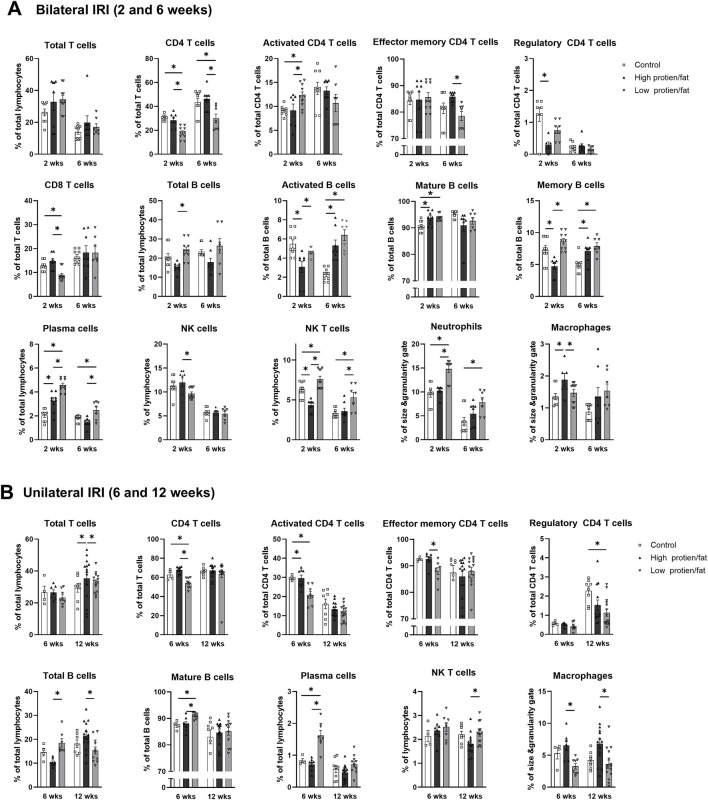
Intrarenal leukocyte subtypes in the post-ischemic kidneys in mice fed low- or high-fat/protein diets. Intrarenal leukocyte subtypes after **(A)** bilateral and **(B)** unilateral ischemia-reperfusion injury. *P < 0.05. Statistical analyses were performed using analysis of variance test followed by Tukey’s test. Activated T cells, CD69^+^ subset; Effector memory T cells, CD44^+^ CD62L-subset; Regulatory CD4 T cells, FoxP3+ CD25^+^ CD4 T cell subset; Activated B cells, CD69^+^ B cell subset; Plasma cells, CD138+ CD126+ subset; Mature B cells, CD21/35 + B cell subset; Memory B cells, CD27^+^ B cell subset.

The number of intrarenal total leukocytes expressing CD45 did not differ between the groups following bilateral or unilateral IRI (Figure 10). The subtypes of intrarenal leukocytes following renal IRI in mice fed high- or low-fat/protein diets are shown in Figure 11. Following bilateral IRI, the high-fat/protein group had fewer regulatory CD4 T, activated B, and NK T cells and more mature B cells, plasma cells, and macrophages than the control group (Figure 11A). The low-fat/protein group had fewer CD4 T and CD8 T cells and more neutrophils, activated CD4 T, mature B, memory B, plasma, and NK T cells than the control group. Following unilateral IRI, the high-fat/protein group had a higher proportion of total T and CD4 T cells than the control group (Figure 11B). The low-fat/protein group had a lower proportion of CD4 T, activated CD4 T, and regulatory CD4 T cells, and a higher proportion of mature B and plasma cells than the control group.

**FIGURE 12:**
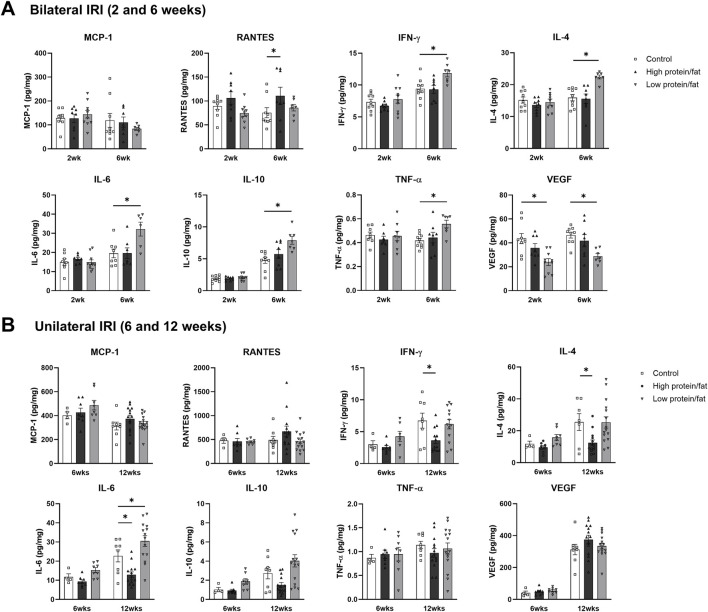
Expression of intrarenal cytokines and chemokines in the post-ischemic kidneys in mice fed low- or high-fat/protein diets. Expression of intrarenal cytokines and chemokines after **(A)** bilateral and **(B)** unilateral ischemia-reperfusion injury. *P < 0.05. Statistical analyses were performed using analysis of variance test followed by Tukey’s test.

Intrarenal cytokine/chemokine levels following renal IRI in mice fed high- or low-fat/protein diets are shown in Figure 12. Following bilateral IRI, the high-fat/protein group showed higher RANTES expression than the control group (Figure 12A). The low-fat/protein group had higher expression of IFN-γ, IL-4, IL-10, IL-6, and TNF-α, and lower expression of VEGF than the control group. Following unilateral IRI, the high-fat/protein group showed lower expression of IFN-γ, IL-4, and IL-6 than the control group, whereas the low-fat/protein group showed higher expression of IL-6 than the control group (Figure 12B).

### 3.7 Effect of sodium, protein, and lipid on the proliferation of hypoxic HK-2 cells

**FIGURE 13:**
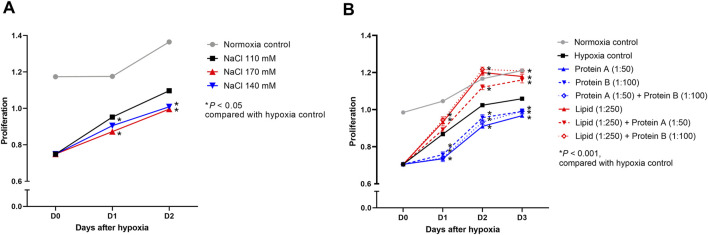
Proliferation of hypoxic HK-2 cells treated with various salt, lipid, and protein concentrations. Proliferation of hypoxic HK-2 cells according to various **(A)** sodium concentrations and **(B)** protein or lipid concentrations in media. *P < 0.05. Statistical analyses were performed using analysis of variance test followed by Tukey’s test.

The proliferation of hypoxic HK-2 cells was inhibited in high sodium-containing media (Na 140 and 170 mmol/L) compared with that of control cells treated with medium containing 110 mmol/L of sodium (Figure 13A). High concentrations of lipid enhanced the proliferation of hypoxic HK-2 cells compared to that of the control cells regardless of the protein concentration (Figure 13B). High concentrations of protein inhibited the proliferation of hypoxic HK-2 cells.

## 4 Discussion

In the present study, we investigated the overall effects of dietary modifications including varying amounts of salt, fat, and protein intake on the healing phase following ischemic AKI. A prolonged low-salt diet during the healing phase after IRI induced proinflammatory changes in the post-ischemic kidney and exacerbated renal tubular damage and fibrosis. Low-fat and low-protein diets during the healing phase after IRI also induced proinflammatory changes in the post-ischemic kidney and impaired healing independent of salt intake. In hypoxic HK-2 cells, high concentrations of sodium and proteins inhibited cell proliferation, whereas high concentration of lipids promoted cell proliferation.

Our study showed that a low-salt diet exacerbates intrarenal inflammation and impedes recovery during the healing phase after renal IRI. Salt intake has been reported to modulate immune cell function, and high salt intake exacerbates intrarenal inflammation (Ito et al., 2023; Wilck et al., 2019). The mechanism by which sodium contributes to inflammation involves the activation of nuclear factor of activated T cells 5 (NFAT5), also known as the TonEBP pathway. NFAT5 is expressed in various tissues and in renal medullary cells. NFAT5 activates downstream inflammatory mediators and represses anti-inflammatory genes (Choi et al., 2020; Wilck et al., 2019). High salt intake has been shown to exacerbate intrarenal inflammation in several animal models of renal failure (Cao et al., 2015; Liu et al., 2021; Oliveira et al., 2020). Previous studies on recovery after renal IRI have shown that high salt intake causes salt-sensitive hypertension and promotes progression to CKD, which may be mitigated by immunosuppression or RAS blockade (Mehrotra et al., 2015; Pechman et al., 2008). In our experiments, high salt intake enhanced intrarenal TGF-β expression and intrarenal leukocytes infiltration after bilateral IRI, and high concentrations of sodium inhibited proliferation of hypoxic HK-2 cells, which is consistent with the findings in previous studies. In contrast, we demonstrated that an absolutely low-salt diet adversely affected recovery during the healing phase. Low-salt diet enhanced intrarenal expression of MCP-1, RANTES, TNF-α, and TGF-β after unilateral IRI. Therefore, the adverse effects of low-salt diet on recovery after IRI may be mediated by proinflammatory changes in the intrarenal immunologic micromilieu and enhanced expression of TGF-β, especially after severe AKI.

In our study, high-fat and high-protein diets during the healing phase after renal IRI were beneficial for renal recovery. A low-fat and low-protein diet may help prevent uremic complications by initially suppressing BUN elevation; however, a low-fat and low-protein diet for more than 2 weeks appeared to result in malnutrition, which inhibits tissue regeneration, prolongs the proinflammatory state, and promotes intrarenal fibrosis. Although our murine data alone did not clearly distinguish the effects of dietary fat and protein modifications, our *in vitro* cell experiments showed that fatty acids promote the proliferation of hypoxic HK-2 cells, suggesting a potentially beneficial effect of a high-fat diet on renal recovery. Long-term high fat intake induces obesity (Hu et al., 2018; Wang et al., 2020), and obesity and metabolic syndrome are well-known risk factors for CKD (Lin et al., 2022; Yoon et al., 2009). Furthermore, high fat intake promotes intrarenal inflammation and injury via various potential mechanisms, such as oxidative stress, mitochondrial dysfunction, and NF-κB activation (Laurentius et al., 2019; Sun et al., 2020). In a renal IRI model, a high-fat diet prior to IRI increased oxidative stress and mitochondrial dysfunction induced by IRI (Prem and Kurian, 2021). However, the effect of fat intake on renal recovery after renal IRI has not been sufficiently investigated. Like metabolically active cardiomyocytes, renal proximal tubular cells also utilize fatty acid oxidation as their primary energy source, resulting in gluconeogenesis rather than glycolysis (Forbes and Thorburn, 2018). In contrast, during renal tubular injury such as IRI, fatty acid oxidation is impaired due to NAD depletion, resulting in increased ATP production via glycolysis (Chen et al., 2024; Simon and Hertig, 2015). In physiological states or chronic illnesses, excess fatty acids may accumulate in tissues and cause lipotoxicity (Katsoulieris et al., 2010). Given the results of the present study demonstrating that a high-fat diet promotes recovery after renal IRI, and fatty acids facilitate the proliferation of hypoxic HK-2 cells, a high-fat diet or fatty acids may improve maladaptive energy metabolism during the healing phase after renal IRI. The effect of high fat intake on renal recovery in patients with ischemic AKI and the underlying mechanisms require further investigation.

Research on optimal protein intake in patients with AKI is limited. Patients with AKI usually exhibit catabolic states and a negative nitrogen balance. The principal goal of nutritional support for AKI includes sufficient supplementation of proteins to maintain metabolic balance, despite little evidence supporting that increasing protein intake can overcome hypercatabolism (Kellum et al., 2012). A high-protein diet is known to cause or aggravate glomerular hyperfiltration (Sällström et al., 2010), consequently contributing to renal cell injury and exacerbating CKD progression (Kanbay et al., 2024). However, the effects of protein intake during the recovery phase of AKI have yet to be determined. Some animal and clinical studies have reported the protective effects of a low-protein diet prior to renal IRI or surgery (Andrews, 1990; Andrews and Bates, 1986; Husain-Syed et al., 2022; Robertson et al., 2015) and no protective effect of preoperative short-term restriction of sulfur-containing amino acids on AKI prevention (Osterholt et al., 2022). However, no study has examined the immunological effects of protein intake during the healing phase of renal IRI. A previous study suggested the protective effect of a high-protein diet through cell cycle arrest and tubular cell protection, as evidenced by increased IGFBP7 and TIMP2 (Husain-Syed et al., 2022). Theoretically, if severely damaged nephrons become dysfunctional, the relatively less damaged nephrons may compensate to a certain extent by facilitating glomerular hyperfiltration, which can be exacerbated by a high-protein diet. The underlying mechanisms of hyperfiltration in AKI may differ from those of hyperfiltration in CKD or diabetes, which are accompanied by tubulo-glomerular feedback and proximal tubular hypertrophy. In the present study, high-fat and high-protein diets did not adversely affect the intrarenal immunologic micromilieu or tubular injury, but the effect of protein intake independent of fat intake could not be determined. Further studies are needed to understand the effect of protein intake on the susceptibility to and recovery from AKI.

Our results emphasize the importance of a balanced nutritional strategy, tailored to each patient’s metabolic demands and clinical condition. Although low-salt and low-protein diets are often recommended in patients with chronic kidney disease (Kellum et al., 2012), those diets are likely to impede recovery from ischemic AKI by triggering proinflammatory responses and promoting renal fibrosis based on our findings. While long-term excessive fat and protein intake leads to adverse effects, including obesity, metabolic syndrome, and glomerular hyperfiltration (Kanbay et al., 2024; Kellum et al., 2012; Sällström et al., 2010), sufficient fat and protein intake may support cell proliferation and tissue regeneration during the recovery phase of ischemic AKI. Therefore, rather than adopting extreme dietary modifications, a balanced approach could be considered with ongoing nutritional modifications and close monitoring of patients’ overall calorie and nutrient balance. Further research is needed to determine the optimal dietary regimens and duration that facilitate AKI recovery.

This study has several limitations. Each diet group was free to consume food without any strict control over their total calorie intake. Dietary modifications can change not only the total calories per unit weight of feed but also the taste of each modified diet. Therefore, our results need to be validated through experiments with controlled total calorie intake. Second, the extreme modifications of the diet composition used in the our study may limit its application in real-world clinical practice. However, we believe that our data reveal the potential effects of dietary modifications on pathophysiological changes in post-ischemic kidneys. Third, *in vitro* models with relatively long-term hypoxic insult and short-term recovery periods compared to *in vivo* IRI models or clinical ischemic AKI may not sufficiently represent the overall metabolic changes in post-ischemic kidneys affected by dietary modifications. Another important limitation was that analyses for innate immune cells were limited in our study. Given the importance of these cells in AKI repair as lymphocytes, future studies using more sophisticated markers for innate immune cells are warranted.

In conclusion, this study demonstrated that excessively low or high salt, low fat, and low protein intake may adversely affect the healing process after renal IRI. Our data suggest that excessively low-salt diet should be avoided, and adequate nutrition, including fat, should be provided to promote renal recovery during the healing phase after ischemic AKI.

## Data Availability

The raw data supporting the conclusions of this article will be made available by the authors, without undue reservation.
